# Executive summary of the guideline for prescribing opioid analgesics for chronic non-cancer pain (third edition) by the Japan Society of Pain Clinicians

**DOI:** 10.1007/s00540-025-03559-x

**Published:** 2025-08-11

**Authors:** Akifumi Kanai, Masako Iseki, Hiroki Iida, Shigeki Yamaguchi, Ayano Oiwa, Hiroshi Yonekura, Narihito Iwashita, Hiroshi Ueno, Yoshiyuki Kimura, Toshifumi Takasusuki, Keisuke Yamaguchi, Shie Iida, Hiroko Ikemiya, Rina Oya, Yoko Sugiyama, Kumiko Tanabe, Ayano Taniguchi, Yoshiyasu Hattammaru, Maki Mizogami, Shinobu Yamaguchi, Keiko Yamada, Sei Fukui

**Affiliations:** 1 Division of Dolorology, Research and Development Center for New Medical Frontiers, Kitasato University School of Medicine, 1-15-1 Kitazato, Minami-ku, Sagamihara City, Kanagawa Prefecture 252-0374 Japan; 2 Department of Anesthesiology and Pain Medicine, Faculty of Medicine, Juntendo University, Tokyo, Japan; 3 Anesthesiology and Pain Relief Center, Central Japan International Medical Center, Minokamo, Japan; 4 Department of Anesthesiology, Dokkyo Medical University School of Medicine, Mibu, Japan; 5 Department of Anesthesiology, Jikei University School of Medicine, Tokyo, Japan; 6 Department of Anesthesiology and Pain Medicine, Fujita Health University Bantane Hospital, Nagoya, Japan; 7 Pain Management Clinic, Shiga University of Medical Science Hospital, Otsu, Japan; 8 Department of Anesthesiology, Kyoto Prefectural University of Medicine, Kyoto, Japan; 9 Department of Anesthesiology and Pain Medicine, Juntendo Tokyo Koto Geriatric Medical Center, Tokyo, Japan; 10 Department of Anesthesiology and Pain Medicine, Gifu University Graduate School of Medicine, Gifu, Japan; 11 Department of Palliative Medicine, Japanese Red Cross Kyoto Daiichi Hospital, Kyoto, Japan; 12 Yoshimura Pain Clinic, Gifu, Japan; 13 Department of Advanced Pain Medicine, Aichi Medical University, Nagakute, Japan

**Keywords:** Chronic pain, Non-cancer pain, Opioid analgesics, Japanese guideline

## Abstract

Opioid analgesics are powerful pain relievers, but inappropriate use can cause a variety of problems. Especially, in chronic non-cancer pain, inappropriate use of opioid analgesics might fail to improve or even worsen the quality of life and activities of daily living of patients impaired by pain. The Japan Society of Pain Clinicians has developed the Guidelines for Prescribing Opioid Analgesics for Chronic Non-cancer Pain (Third Edition), which answers basic clinical questions concerning opioid management as summary statements in accordance with the most recent scientific evidence and expert opinion. The guidelines emphasize patient selection (e.g., obvious organic cause of the persistent pain, low risk of psychosocial factors, pain refractory to non-opioid therapies, and good adherence to medication). Furthermore, patients should be treated with an oral dose of 90 morphine milligram equivalents/day as the upper limit and target duration of 3–6 months. For pain in cancer survivors that is not directly caused by cancer, such as pain after treatment or pain associated with complications or pre-existing conditions, these guidelines should be followed.

## Introduction

As Japan's society ages, interest in healthy life expectancy as well as average life expectancy is increasing. The importance of maintaining quality of life (QOL) and activities of daily living (ADLs) is being emphasized. Pain not only significantly impairs patients' QOL and ADLs, but can also lead to social problems such as loss of productivity and increased medical costs. The Japan Society of Pain Clinicians has developed various guidelines to appropriately respond to pain. One example of such guidelines is the Guidelines for Prescribing Opioid Analgesics for Chronic Non-Cancer Pain. Opioid analgesics are powerful pain relievers, but inappropriate use can cause a variety of problems. Especially, in chronic non-cancer pain, inappropriate use of opioid analgesics might fail to improve or even worsen the QOL and ADLs of patients with pain. In Japan, chronic non-cancer pain was added as an indication for some opioid analgesics in 2011. Opioid analgesic prescriptions for chronic non-cancer pain had officially begun, and they are becoming more widespread in pain medicine. Opioid analgesics are expected to continue as an option for improving the QOL and ADLs of patients with chronic non-cancer pain. With these considerations in mind, the first edition of these guidelines was developed and published in 2012.

The situation has changed over the years since 2012, when the First Edition was prepared, to 2017, when the revised Second Edition was prepared, and to the present. The following points were considered by the working group when they began working on the revision of these guidelines.

**Fig. 1:**
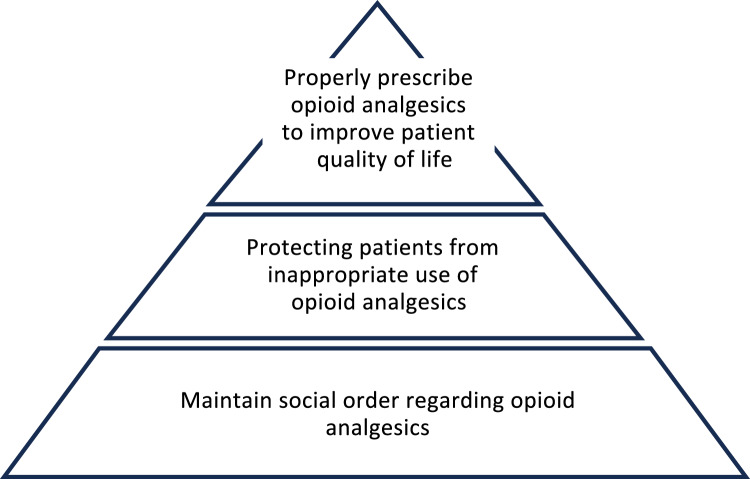
Outline of the guidelines

1. In Europe and the United States, where prescribing opioid analgesics for chronic non-cancer pain was the norm, it has become clear that long-term or high-dose prescribing is associated with a lack of evidence regarding efficacy. In fact, it poses various problems and guidelines have been revised in other countries.
2. Inappropriate use of opioid analgesics in Japan has become a problem, especially among cancer survivors who have relatively easy access to opioid analgesics.
3. With the increase in the number of opioid analgesics and related drugs available in Japan and the launch of generic versions of some opioid analgesics, the indications and instructions about dosage and administration in package inserts must be more closely followed.
4. In some countries, the social flooding of opioid analgesics due to easily accessible prescriptions has led to serious social problems, known as the opioid crisis. For this reason, it is necessary to continue to regulate and control opioid analgesics in Japan. In light of the current situation in Japan, the Japan Society of Pain Clinicians has determined an urgent need to revise the guidelines. It has appointed new committee members to form the Working Group (WG) for Guidelines for Prescribing Opioid Analgesics for Chronic non-cancer Pain, which has begun work on the revised Third Edition. As with the revised Second Edition, the most important messages of the First Edition is maintained: “Protect the social order with respect to opioid analgesics,” “Protect patients from inappropriate use of opioid analgesics,” and “Improve patients' quality of life with opioid analgesics.” These three basic principles that were the most important in the first edition (Fig. 1) have been revised to take into account the current situation in Japan and trends overseas.

In revising these guidelines, we have tried to ensure consistency with other treatment guidelines and statements produced by the society. To make these guidelines more publicly accessible, we have created them in the form of clinical questions (CQs) in accordance with the basic policy of evidence-based medicine (EBM) and the Guidelines Promotion Project (Minds) by the Japan Council for Quality Health Care.

### Level of evidence

The level of evidence was determined based on the third edition of the Minds Manual for Guideline Development 2020 [1] and the GRADE System for Clinical Practice Guidelines, third edition [2].

The overall strength of evidence for each CQ or the outcome in general was defined as follows.

A (high): We are very confident that the true effect lies close to that of the estimate of the effect.
B (moderate): We are moderately confident in the effect estimate.
C (low): We have limited confidence in the effect estimate.
D (very low): We have very little confidence in the effect estimate.

### Determining the level of recommendation

Four factors were considered when determining the level of recommendation: overall certainty of evidence, balance between desirable and undesirable effects, values and preferences, and cost and resource use. The strength of recommendation was specified and presented as (1) strong or (2) weak.

If it was not possible to make a clear recommendation due to reasons such as being unable to determine the strength of the recommendation, the recommendation level was not stated.

The certainty of the evidence (A, B, C, or D) and the strength of the recommendation (1 or 2) were combined in each statement.

Examples:

1. Strongly recommend treatment I for disease P (1A)
  = (strong recommendation, based on strong evidence)
2. Weakly recommend treatment I for disease P (2C)
  = (weak recommendation, based on weak evidence)
3. Strongly recommend against treatment I for disease P (1B)
  = (strong recommendation, based on moderate evidence).

### Criteria for recommendations and consensus building

A draft version of this document was distributed to each WG member for review and comments. WG members were asked to confirm the text. After feedback was received, a consensus-building meeting was held with all members in attendance. Consensus on each proposed recommendation was reached when at least 80% of members agreed. Discussions and revisions continued until the consensus criteria were met.

The present article summarizes the recommendations in the Guidelines for Prescribing Opioid Analgesics for Chronic Non-cancer Pain, Third Edition, published by the Japan Society of Pain Clinicians in 2024 (Table 1). Readers interested in the topic are encouraged to refer to the full guidelines to gain a more complete understanding of the appropriate context for the recommendations.

**Table 1:** Clinical questions in the guidelines for prescribing opioid analgesics for chronic non-cancer pain, third edition by the Japanese Society of Pain Clinicians

I. What are opioids?
Clinical question 1-1: What are opioids?
Clinical question 1-2: What are opioid receptors?
Clinical question 1-3: What are opioid analgesics?
Clinical question 1-4: What are strong opioid analgesics and weak opioid analgesics?
Clinical question 1-5: What are medical narcotics?
II. Opioid analgesics: specifics
Clinical question 2-1: What kind of opioid analgesic is codeine phosphate?
Clinical question 2-2: What kind of opioid analgesic is tramadol?
Clinical question 2-3: What kind of opioid analgesic is a buprenorphine?
Clinical question 2-4: What kind of opioid analgesic is morphine hydrochloride?
Clinical question 2-5: What kind of opioid analgesic is a fentanyl?
Clinical question 2-6: What kind of opioid analgesic is oxycodone hydrochloride?
III. Treatment with opioid analgesics
Clinical question 3-1: Are opioid analgesics useful for chronic non-cancer pain?
Clinical question 3-2: Which patients with chronic non-cancer pain can benefit from opioid analgesics?
Clinical question 3-3: What is the purpose of treatment with opioid analgesics for chronic non-cancer pain?
Clinical question 3-4: Which opioid analgesics can be prescribed to patients with chronic non-cancer pain in Japan?
Clinical question 3-5: What types of opioid analgesics are suitable for chronic non-cancer pain?
Clinical question 3-6: When is treatment with opioid analgesics is indicated for chronic non-cancer pain?
Clinical question 3-7: Which patients with chronic non-cancer pain should avoid treatment with opioid analgesics?
Clinical question 3-8: How should the use of opioid analgesics be considered for sudden exacerbation of pain in patients with chronic non-cancer pain?
Clinical question 3-9: Is opioid switching useful for chronic non-cancer pain?
IV. Initiating treatment with opioid analgesics
Clinical question 4-1: What patient assessments are necessary when starting treatment with opioid analgesics?
Clinical question 4-2: What should be considered when starting treatment with opioid analgesics?
Clinical question 4-3: Is written explanation and consent useful for appropriate treatment with opioid analgesics?
Clinical question 4-4: What is the optimal initial dose of opioid analgesics?
Clinical question 4-5: How is the optimal dose of opioid analgesics determined (appropriate dose adjustment)?
Clinical question 4-6: What is the appropriate follow-up interval during treatment with opioid analgesics?
Clinical question 4-7: What is the optimal dosage of opioid analgesics?
Clinical question 4-8: Should the use of opioid analgesics be restricted by setting the treatment period?
V. Side effects of opioid analgesics
Clinical question 5-1: What should be considered during treatment with opioid analgesics?
Clinical question 5-2: What are side effects of opioid analgesics?
Clinical question 5-3: How are nausea and vomiting caused by opioid analgesics managed?
Clinical question 5-4: How is opioid-induced constipation managed?
Clinical question 5-5: Are peripheral μ-opioid receptor antagonists effective for opioid-induced constipation?
Clinical question 5-6: How is drowsiness caused by opioid analgesics managed?
Clinical question 5-7: What adverse events are associated with long-term use of opioid analgesics?
VI Discontinuation of treatment with opioid analgesics
Clinical question 6-1: When should treatment with opioid analgesics be discontinued?
Clinical question 6-2: How should opioid analgesics be reduced or discontinued?
Clinical question 6-3: What are the characteristics of patients who are likely to be treated with opioid analgesics for a long time or at high doses?
Clinical question 6-4: How can patients who have been treated with opioid analgesics for a long time or on a higher dose be managed?
VII. Inappropriate use of opioid analgesics
Clinical question 7-1: What is abuse of opioid analgesics?
Clinical question 7-2: What is physical dependence on opioid analgesics?
Clinical question 7-3: What is the nature of psychological dependence on opioid analgesics?
Clinical question 7-4: What are the symptoms of opioid withdrawal?
Clinical question 7-5: What are the characteristics of patients who are prone to inappropriate use of opioid analgesics?
Clinical question 7-6: How should inappropriate use of opioid analgesics be assessed?
Clinical question 7-7: How should patients who have been using opioid analgesics inappropriately be managed?
Clinical question 7-8: How is opioid analgesic overdose (respiratory depression) treated?
VIII. Indications for treatment with opioid analgesics
Clinical question 8-1: Is treatment with opioid analgesics effective for chronic low back pain?
Clinical question 8-2: Is treatment with opioid analgesics effective for osteoarthritis (OA)?
Clinical question 8-3: Is treatment with opioid analgesics effective for compression fracture pain?
Clinical question 8-4: Is treatment with opioid analgesics effective for herpes zoster-related pain?
Clinical question 8-4-1: Is tramadol effective for post-herpetic neuralgia (PHN)?
Clinical question 8-4-2: Are strong opioid analgesics effective for post-herpetic neuralgia (PHN)?
Clinical question 8-5: Is treatment with opioid analgesics effective for painful diabetic neuropathy (PDN)?
Clinical question 8-6: Is treatment with opioid analgesics effective for chronic post-surgical pain (CPSP)?
Clinical question 8-7: Is treatment with opioid analgesics effective for post-spinal cord injury pain?
Clinical question 8-8: Is treatment with opioid analgesics effective for phantom limb pain (PLP)?
Clinical question 8-9: Is treatment with opioid analgesics effective for complex regional pain syndrome (CRPS)?
Clinical question 8-10: Is treatment with opioid analgesics effective for thalamic pain?
Clinical question 8-11: Is treatment with opioid analgesics effective for chemotherapy-induced peripheral neuropathy (CIPN) pain?
Clinical question 8-12: Are there conditions for which opioid analgesics are not recommended in principle?
IX. Prescription of opioid analgesics in special circumstances
Clinical question 9-1: What are the considerations for prescribing opioid analgesics to pregnant patients?
Clinical question 9-2: What are the considerations for prescribing opioid analgesics to elderly patients?
Clinical question 9-3: What are the considerations for prescribing opioid analgesics to patients with impaired renal function?
Clinical question 9-4: What are the considerations for prescribing opioid analgesics to patients with impaired liver function?
Clinical question 9-5: What are the considerations for prescribing opioid analgesics to patients with sleep apnea syndrome (SAS)?
Clinical question 9-6: What are the considerations for prescribing opioid analgesics to patients with work-related injuries and traffic accidents?
Clinical question 9-7: What are the considerations for prescribing opioid analgesics to adolescent and young adults (AYAs)?
X. Other
Clinical question 10-1: Is the combination of opioid analgesics and adjuvant analgesics effective?
Clinical question 10-2: What are the considerations for patients being treated with opioid analgesics who want to drive?
Clinical question 10-3: What are the considerations when traveling abroad during treatment with opioid analgesics?
Clinical question 10-4: What are the limitations of the guidelines for prescribing opioid analgesics for chronic non-cancer pain?

## Purpose of presenting this paper

This manuscript is a translation of the guideline originally published in Japanese. Although the content has already been made publicly available on the website of the Japan Society of Pain Clinicians in Japanese, the purpose of this English publication is to communicate the importance of opioid management in chronic non-cancer pain to an international audience.

In some countries, the widespread availability of opioid prescriptions has led to serious social issues, known as the "opioid crisis." Therefore, in this article, we would like to present how opioid analgesics are appropriately regulated and managed in Japan.

### I. What are opioids?

#### Clinical question 1-1: What are opioids [3]?

##### Summary statement

Opioids are defined as “a general term for substances that have an affinity for the receptors to which opium binds (opioid receptors).”

#### Clinical question 1-2: What are opioid receptors [3]?

##### Summary statement

Opioid receptors are a group of G protein-coupled receptors with opioids as ligands.

Their activation affects various intracellular signaling pathways. They exert various effects, including an analgesic effect, by inhibiting nociceptive transmission and activating the descending pain pathway.

#### Clinical question 1-3: What are opioid analgesics [3]?

##### Summary statement

Opioid analgesics are medications used to relieve chronic non-cancer pain. Non-opioid analgesics (mainly nonsteroidal anti-inflammatory drugs and acetaminophen) and adjuvant analgesics (mainly antidepressants and gabapentinoids) are also used to relieve chronic non-cancer pain. Opioid analgesic is a general term for a drug that exerts its analgesic effect via opioid receptors.

#### Clinical question 1-4: What are strong opioid analgesics and weak opioid analgesics [4]?

##### Summary statement

The Guidelines for the Pharmacologic Management of Neuropathic Pain, Second Edition, by the Japan Society of Pain Clinicians classifies opioid analgesics into mild opioid analgesics, moderate opioid analgesics, and strong opioid analgesics. The Third Edition of the guidelines also originally classified them into three categories. However, the new guidelines from the World Health Organization distinguish between strong opioid analgesics and weak opioid analgesics, and refer to each of them separately. Accordingly, the final version of the Third Edition of the guidelines also classifies opioid analgesics as strong or weak.

#### Clinical question 1-5: What are medical narcotics [5]?

##### Summary statement

The term narcotic refers to a class of drugs derived from opium or opium-like compounds that have a powerful analgesic effect that also cause marked changes in mental and behavioral states and have the potential for dependence and tolerance. Usually, medical narcotics include synthetic or natural drugs such as pethidine as well as fentanyl and its derivatives. Although ketamine and cocaine are not opioids, they are designated as medical narcotics.

### II. Opioid analgesics: specifics (Table 2)

**Table 2:** Various opioid analgesics

Drug name	Metabolizing enzymes	Active metabolites	Product names	Indication for chronic non-cancer pain	Control classification
Codeine	CYP2D6, 3A4, UGT	Morphine	Codeine phosphate		
			1% powder, 5 mg tablet	+	None
			API, 10% powder, 20 mg tablet	+	Narcotic
Tramadol	CYP2D6, 3A4, 2B6	M1	Tramal® (immediate-release tablet)	+	None
			Twotram® (immediate / extended-release combination tablet)	+	None
			Onetram® (extended-release tablet)	+	None
			Tramcet® (tramadol / acetaminophen combination tablet)	+	None
			Injection	-	None
Buprenorphine	CYP3A4, UGT	Norbuprenorphine	Norspan® (patch)	+	Psychotropic
			Suppository, injection	-	Psychotropic
Morphine	UGT	Morphine-6-glucuronide	Tablet, bulk	+	Narcotic
			Suppository, oral solution	-	Narcotic
			All long-acting opioid analgesics	-	Narcotic
			Injection	+	Narcotic
Fentanyl	CYP3A4	-	Patch for 3 days	+ (Original medicines only)	Narcotic
			Patch for 1 days	+ (Original medicine only)	Narcotic
			Sublingual tablet, transmucosal formations	-	Narcotic
			Injection	+	Narcotic
Oxycodone	CYP3A4, 2D6	Noroxymorphone	Short-acting tablet, powder, oral solution	-	Narcotic
		Oxymorphone	Long-acting (extended-release) tablet	+ (OxyContin® TR only)	Narcotic
			Injection	-	Narcotic

*CYP* cytochrome P450, *UGT* UDP-glucuronosyl transferase, *M1* mono-O-desmethyltramadol, *API* active pharmaceutical ingredient

#### Clinical question 2-1: What kind of opioid analgesic is codeine phosphate [6–20]?

##### Summary statement

Codeine, which is metabolized by the liver into morphine, exerts an opioid analgesic effect. Although the analgesic effect of codeine is about 1/6 that of morphine, there are large variations in its analgesic effect across individuals, because codeine is metabolized by CYP2D6 into morphine. In addition, morphine-6-glucuronide (M6G), a metabolite of morphine, is also active, so there is a risk that the effect will be enhanced and prolonged in patients with impaired renal function. Codeine is effective for chronic non-cancer pain. Like morphine, it has side effects such as constipation, nausea, and drowsiness.

#### Clinical question 2-2: What kind of opioid analgesic is tramadol?

##### Summary statement [6, 21–34]

Tramadol has a direct analgesic effect due to the inhibition of the reuptake of serotonin and noradrenaline. Its metabolite mono-O-desmethyl tramadol (M1) partially activates the μ-opioid receptor. Since M1 is produced by the enzyme CYP2D6, which has individual and racial differences in activity, there are large variations in the opioid analgesic effect of tramadol across individuals. It is effective for nociceptive and neuropathic pain, but tramadol is thought to be more effective for neuropathic pain.

#### Clinical question 2-3: What kind of opioid analgesic is buprenorphine? [35–55]

##### Summary statement

Buprenorphine is a full agonist that acts in a dose-dependent manner on the μ-opioid receptor. It is a partial agonist that has a ceiling effect on respiratory depression even when the dose is increased. When used in combination with other full agonists of the μ-opioid receptor, the analgesic effect of buprenorphine is enhanced and respiratory depression tends to be antagonized. In addition, because it antagonizes κ-opioid receptors, it has strong anti-hyperalgesic and antidepressant effects. Tolerance, abuse, fatal overdose, endocrine dysfunction, and immune dysfunction are relatively rare.

#### Clinical question 2-4: What kind of opioid analgesic is morphine hydrochloride [56–66]?

##### Summary statement

Morphine has been used in clinical practice for a long time. It is a strong opioid analgesic with a wealth of evidence on various routes of administration. Because it is water-soluble, it is not easily absorbed by fat and has a low blood transfer rate when administered into the epidural space or subarachnoid space. Thus, it has a strong and long-lasting analgesic effect. When morphine is administered systemically, its metabolite M6G (10–15% of morphine) has the same analgesic effect as morphine. Since M6G can be excreted in the urine without being metabolized, it has a long duration of action. In patients with renal dysfunction, the analgesic effect and side effects are both enhanced.

#### Clinical question 2-5: What kind of opioid analgesic is fentanyl [60, 65, 67–75]?

##### Summary statement

Fentanyl is a strong opioid analgesic that is fat-soluble. While it is more potent and has a faster onset of action than water-soluble morphine, it has a shorter duration of action and is more likely to cause respiratory depression. Transdermal patches make it easy to achieve a stable blood concentration and constant analgesic effect. However, when the patient has a fever, the absorption of fentanyl is accelerated and its blood concentration increases; also caution is required when using it in combination with CYP3A4 inhibitors or psychotropic drugs that increase side effects. Only fentanyl transdermal patches in the original formulation are covered by health insurance for chronic non-cancer pain. Qualifications for prescribing fentanyl patches can be obtained after a confirmation letter is issued for taking an e-learning course.

#### Clinical question 2-6: What kind of opioid analgesic is oxycodone hydrochloride [13, 76–87]?

##### Summary statement

Oxycodone is a strong opioid analgesic that is water-soluble. Its analgesic effect is the same as that of morphine, but because its bioavailability is high, it is possible to obtain the same analgesic effect with two-thirds of the dose when administered orally. The main metabolic enzymes are CYP3A4/5 and CYP2D6. Active metabolites such as noroxymorphone have a long duration of action. The unchanged drug is also excreted in the urine in relatively large amounts. Caution, albeit less than for morphine, is required when using it in patients with impaired renal function. Oxycodone extended-release formulation (anti-tampering agent) is covered by insurance for chronic non-cancer pain. Qualifications for prescribing oxycodone hydrochloride can be obtained after a confirmation letter is issued for taking an e-learning course.

### III. Treatment with opioid analgesics

#### Clinical question 3-1: Are opioid analgesics useful for chronic non-cancer pain [88–94]?

##### Recommendations

For the treatment of chronic non-cancer pain, we weakly recommend pharmacological treatment with a variety of non-opioid analgesic agents. We recommend prioritizing non-pharmacological treatments. Opioid analgesics could be prescribed only when the benefits of relieving pain and physical function are deemed to outweigh the risks of opioid analgesics.**【2C】**

##### Summary statement

For the treatment of chronic non-cancer pain, pharmacological therapy with a variety of non-opioid analgesic agents and non-pharmacological therapy should be prioritized. Opioid analgesics should only be prescribed when the beneficial effects on pain and physical function improvement are deemed to outweigh the risks of opioid analgesics use. In such cases, it is also important to appropriately combine it with non-opioid analgesic agents, non-pharmacological therapy, or both.

#### Clinical question 3-2: Which patients with chronic non-cancer pain can benefit from opioid analgesics? [88, 93, 94]

##### Recommendations

For patients with chronic non-cancer pain who do not currently or have not previously had a substance use disorder and who do not have other active psychiatric disorders, we weakly recommend that they be given a trial of opioid analgesics if their pain persists despite optimal non-opioid pharmacological treatment.**【2B】**

##### Summary statement

When using opioid analgesics for chronic non-cancer pain, it should be a part of multi-modal analgesia that includes non-pharmacological therapy and pharmacological therapy with non-opioid analgesics. Opioid analgesics should not be used alone.

#### Clinical question 3-3: What is the purpose of treatment with opioid analgesics for chronic non-cancer pain? [88, 95]

##### Summary statement

The aim of treatment with opioid analgesics for chronic non-cancer pain is to alleviate pain without causing a deterioration in the patient's QOL due to adverse events and to improve QOL that has been diminished by pain. The goal of pain relief should be set before the administration of opioid analgesics and shared by the medical staff and the patient.

#### Clinical question 3-4: Which opioid analgesics can be prescribed to patients with chronic non-cancer pain in Japan [96]?

##### Summary statement

The opioid analgesics that can be prescribed to patients with chronic non-cancer pain in Japan are tramadol, codeine phosphate, buprenorphine transdermal patch, immediate-release morphine hydrochloride powder and tablets, opium, pethidine, fentanyl transdermal patch, and modified-release oxycodone (modified-release formulation). However, some of these medications are restricted to certain formulations. The Act on Securing Quality, Efficacy and Safety of Products including Pharmaceuticals and Medical Devices and the content of the documents for each drug regulated based on this act must be observed.

#### Clinical question 3-5: What types of opioid analgesics are suitable for chronic non-cancer pain [88, 97, 98]?

##### Summary statement

When starting treatment with opioid analgesics for chronic non-cancer pain, consider using a fast-acting weak opioid analgesic such as tramadol to avoid the risk of unexpected overdose. On the other hand, when continuing treatment, formulations and routes of administration that have a lower risk of opioid use disorder should be selected.

(Fig. 2).

**Fig. 2:**
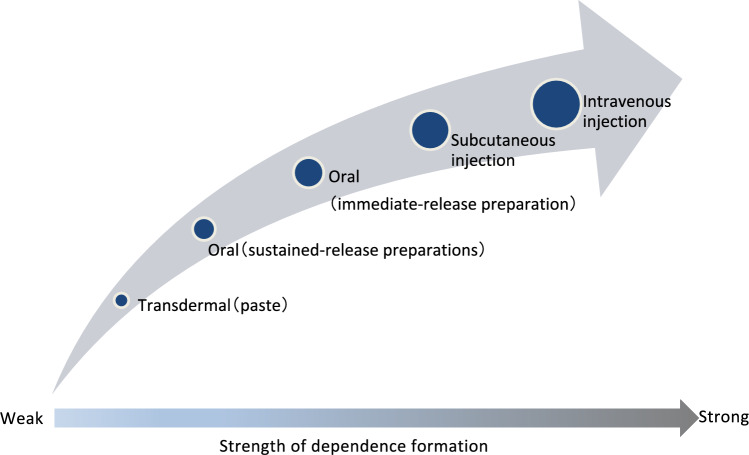
Formation of psychiatric dependence on opioids in relation to formulation and route of administration

#### Clinical question 3-6: When is treatment with opioid analgesics indicated for chronic non-cancer pain [99, 100]?

##### Summary statement

Treatment with opioid analgesics for chronic non-cancer pain should only be considered if the benefits to the patient in terms of pain relief and ADLs outweigh the risks.

#### Clinical question 3-7: Which patients with chronic non-cancer pain should avoid treatment with opioid analgesics [99, 101–104]?

##### Summary statement

Patients with chronic non-cancer pain who have the following characteristics should avoid treatment with opioid analgesics or treated with extreme care:

1. Substance abuse disorder such as alcoholism or illicit drug abuse and overdose.
2. Mental health condition such as depression or anxiety disorder, or taking benzodiazepines.
3. Age 65 years or older.
4. Pregnancy.
5. Moderate-to-severe sleep apnea syndrome.
6. Renal or hepatic dysfunction.

#### Clinical question 3-8: How should the use of opioid analgesics be considered for sudden exacerbation of pain in patients with chronic non-cancer pain [105]?

##### Summary statement

Opioid analgesics should not be readily used for sudden exacerbations of pain that occur in patients with chronic non-cancer pain. In addition, the use of opioid analgesics as a rescue treatment during treatment with opioid analgesics is not recommended, as it is likely to lead to an increase in the overall dose and abuse. Assessment of pain, use of non-opioid analgesics, and self-management with non-pharmacologic therapies are recommended.

#### Clinical question 3–9: Is opioid switching useful for chronic non-cancer pain [89]?

##### Recommendations

In patients with chronic non-cancer pain who are using opioid analgesics, if the adverse effects continue despite appropriate management or the target level of analgesic effect is not achieved despite appropriate dose escalation, switching to another opioid analgesic rather than continuing with the currently used opioid is recommended.【**2C】**

##### Summary statement

Opioid switching refers to the process of changing from one opioid analgesic to another when the side effects of the current opioid analgesic make continued administration undesirable, or the analgesic effect is insufficient (Table 3).

**Table 3:** Equivalent strength conversion table for opioid analgesics available in Japan for chronic non-cancer pain

Oral morphine HCl (mg/day)	Oral tramadol (mg/day)	Oral codeine phosphate (mg/day)	Oxycontin^Ⓡ^ TR Tablets (mg/day)	NORSPAN^Ⓡ^tape (mg/day)	Fentos_Ⓡ_ tape (mg/day)	OneDuro^Ⓡ^patch (mg/day)	Durotep^Ⓡ^MT Patch (mg/day)
30	150	180	20	20	1	0.84	2.1
60	300		40		2	1.7	4.2
90			60		3 (1 + 2)	2.54 (0.84 + 1.7)	6.3 (2.1 + 4.2)

### IV. Initiating treatment with opioid analgesics

#### Clinical question 4-1: What patient assessments are necessary when starting treatment with opioid analgesics [99, 106]?

##### Summary statement

When starting treatment with opioid analgesics, it is most important to exclude patients at high risk of dependence and abuse through careful history-taking and examination. In addition, because strict control of opioid analgesic use is also required on a regular basis after treatment has started, patients who do not follow the doctor's instructions or patients with extremely poor cognitive function and poor medication adherence are not eligible for treatment. Opioid analgesics should be avoided in patients with suspected psychosocial factors contributing to persistent pain and in patients with psychiatric disorders.

#### Clinical question 4-2: What should be considered when starting treatment with opioid analgesics [99, 100, 106–108]?

##### Summary statement

The aim of using opioid analgesics for chronic non-cancer pain is not to eliminate the pain quickly, but to improve QOL and ADLs by alleviating pain. It is important to clearly recognize this treatment goal. This awareness must also be shared by medical staff and patients.

#### Clinical question 4-3: Is written explanation and consent useful for appropriate treatment with opioid analgesics [109–115]?

##### Recommendations

We suggest using a treatment consent form to establish an informed consent process for the use of opioid analgesics. This helps clarify expectations for both the patient and the physician and provides a clear understanding of the nature of opioid analgesic treatment, including endpoints, goals, and strategies for when treatment does not progress as intended.**【2D】**

##### Summary statement

Treatment with opioid analgesics should not be initiated without careful consideration. In particular, for strong opioid analgesics, it is necessary to provide a clear explanation and obtain consent that details the risks associated with treatment, responsibilities and compliance requirements of healthcare professionals and patients, and shared understanding of treatment goals. When initiating treatment with weak opioid analgesics such as tramadol, preparing a written explanation and consent form is not mandatory. However, the same principles of explanation and consent are applicable (Tables 4 and 5).

**Table 4:**
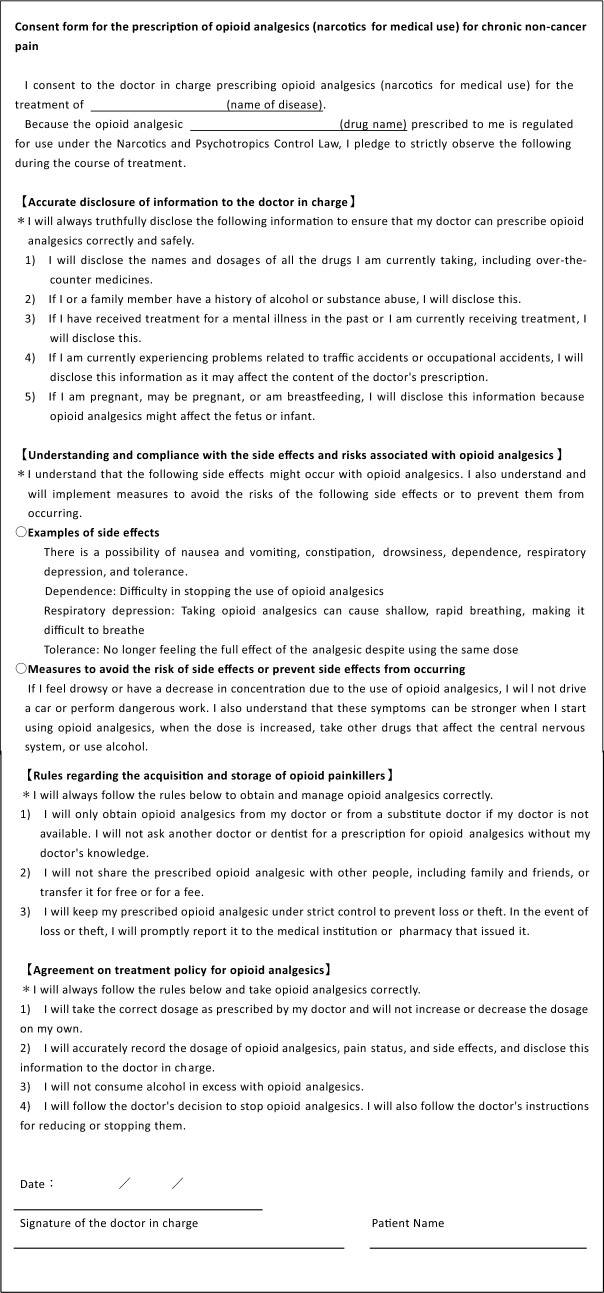
Example of a consent form for the prescription of opioid analgesics for chronic non-cancer pain

**Table 5:** Elements to be explained when obtaining written informed consent

【Compliance with treatment guidelines for opioid analgesics】
When starting opioid analgesics for the treatment of chronic non-cancer pain, the following items should be explained to the patient and a consent form completed
(1) The ultimate goal of opioid analgesic treatment is not solely pain relief, but rather an improvement in quality of life
(2) Clarify and ensure that the patient understands the purpose of opioid analgesic treatment
(3) The physician makes decisions regarding the type of opioid analgesic to be used, when to start using it, how to adjust the dosage, and when to stop using it. The patient is not allowed to change these usage characteristics on their own
(4) Opioid analgesics can cause a variety of side effects
(5) While using opioid analgesics, regular medical examinations at intervals set by the doctor are necessary
(6) Do not receive prescriptions for opioid analgesics from multiple medical facilities at the same time
(7) Opioid analgesic treatment is not intended to be lifelong
(8) Never give opioid analgesics to other people
(9) Unused opioid analgesics that are no longer needed should be promptly taken to the medical institution or pharmacy that issued them

#### Clinical question 4-4: What is the optimal initial dose of opioid analgesics [115]?

##### Summary statement

Opioid analgesics should be initiated at low doses. Prescriptions should be kept to the minimum effective dose.

#### Clinical question 4–5: How is the optimal dose of opioid analgesics determined (appropriate dose adjustment) [94, 99, 115–117]?

##### Summary statement

The optimal dose of opioid analgesics for chronic non-cancer pain should be individually adjusted based on a clinical evaluation of pain severity, its impact on daily life, and other relevant factors.

#### Clinical question 4–6: What is the appropriate follow-up interval during treatment with opioid analgesics?

##### Summary statement

After initiating opioid analgesics in an outpatient setting, shorter intervals of approximately 7–14 days between follow-up visits are recommended to assess treatment effects.

#### Clinical question 4-7: What is the optimal dosage of opioid analgesics [99]?

##### Recommendations

It is recommended that patients should be treated with oral opioid analgesics at 60 morphine milligram equivalents/day or less. If more opioids are necessary, careful administration is needed, considering the benefits and risks to the patient. It is highly recommended that the maximum dose should not exceed 90 morphine milligram equivalents/day.【1B】

##### Summary statement

These guidelines recommend treatment with opioid analgesics at 60 morphine milligram equivalents/day or less. If more opioids are necessary, careful administration is needed, considering the benefits and risks to the patient. It is highly recommended that the maximum dose should not exceed 90 morphine milligram equivalents/day.

#### Clinical question 4-8: Should the use of opioid analgesics be restricted by setting the treatment period [89]?

##### Recommendations

The effectiveness of a strong opioid analgesic on chronic non-cancer pain should be evaluated within 1 month from initiation of treatment and usually continued for 3 months. Consider stopping or reducing opioids 6 months after initiation of treatment, even if pain is well controlled.**【2C】**

##### Summary statement

The decision to continue or stop potent opioid therapy should occur at 1 month after its effectiveness has been confirmed. Treatment will generally last for 3 months. It is weakly recommended that after 6 months of opioid treatment with a good response, withdrawal or dose reduction be considered with the patient.

Although tramadol, a weak opioid analgesic, is excluded from this recommendation, unnecessary long-term administration should be avoided; the need should be continuously re-examined.

### V. Side effects of opioid analgesics

#### Clinical question 5-1: What should be considered during treatment with opioid analgesics [99, 118]?

##### Summary statement

During treatment with opioid analgesics, attention must be paid to side effects, such as nausea, vomiting, constipation, and drowsiness as well as inappropriate use such as abuse and dependence. Manage the dosage of opioid analgesics, so that it does not become high or administered for long periods of time.

#### Clinical question 5-2: What are side effects of opioid analgesics [119, 120]?

##### Summary statement

Nausea, vomiting, constipation, and drowsiness occur frequently at the start of treatment with opioid analgesics. Other symptoms include dizziness, delirium, and urinary disorders. Although rare, respiratory depression can occur with overdose. In addition, it has been reported that long-term use can cause hypersensitivity and gonadal dysfunction.

#### Clinical question 5-3: How are nausea and vomiting caused by opioid analgesics managed [121, 122]?

##### Recommendation

For opioid-induced nausea and vomiting, considering a change in the route of administration and opioid switching are weakly recommended.**【2D】**

##### Summary statement

Nausea and vomiting are likely to occur when treatment with opioid analgesics is started; countermeasures are necessary. Due to the development of tolerance, nausea and vomiting often improve in approximately 1–2 weeks, but countermeasures are necessary again when the dose of opioid analgesics is increased.

#### Clinical question 5-4: How is opioid-induced constipation managed [123]?

##### Recommendation

We strongly recommend ongoing measures such as laxatives throughout the treatment period with opioid analgesics.**【1B】**

##### Summary statement

Constipation occurs frequently after starting treatment with opioid analgesics.

Since tolerance rarely develops, it is necessary to take continuous measures such as laxative administration throughout the treatment period.

#### Clinical question 5-5: Are peripheral μ-opioid receptor antagonists effective for opioid-induced constipation [124–133]?

##### Recommendation

We strongly recommend the use of peripheral μ-opioid receptor antagonists for opioid-induced constipation that does not improve with general laxatives.**【1B】**

##### Summary statement

Peripheral μ-opioid receptor antagonists are effective for opioid-induced constipation. They are also effective for opioid-induced constipation that does not improve with general laxatives. The main side effects are diarrhea, abdominal pain, and nausea (Tables 6 and 7).

**Table 6:** Various μ-opioid receptor antagonists

Common name	Approval status in Japan	Formulation (dose)	Efficacy and effects	Dosage and administration
Naloxegol	Not approved	Tablet (12.5 mg, 25 mg)	【USA】Opioid analgesic-induced constipation in patients with chronic non-cancer pain	【USA】25 mg once a day
			【Europe】Opioid-induced constipation with insufficient laxative effect	【Europe】25 mg once a day
Methyl-naltrexone	Not approved	Tablet (150 mg) Injection (8 mg, 12 mg)	【USA (tablets, injection) 】 1. Opioid-induced constipation in patients with chronic non-cancer pain 2. Opioid-induced constipation in patients with advanced disease who are not responding adequately to laxatives	【USA】1. 450 mg (tablet) once a day in the morning or 12 mg once a day subcutaneously 2. Subcutaneous administration once a day, based on the weight conversion table
			【Europe (injection) 】 Opioid-induced constipation with insufficient laxative effect	【Europe】(patients not in palliative care) 12 mg once daily subcutaneously 4–7 days a week, (palliative care patients) 8 or 12 mg once every 2 days subcutaneously depending on body weight
Alvimopan	Not approved	Capsule (12 mg)	Shortening of the recovery time for upper and lower gastrointestinal motility after surgery involving partial resection of the intestines with primary anastomosis	【USA】12 mg orally 30 min to 5 h before surgery, and 12 mg orally twice a day for up to 7 days from the day after surgery until discharge
Naldemedine	Approved	Tablet (0.2 mg)	【USA】Opioid-induced constipation in patients with chronic non-cancer pain	【USA】0.2 mg once a day
			【Europe】Opioid-induced constipation treated with laxatives	【Europe】0.2 mg once a day
			【Japan】Opioid-induced constipation	【Japan】0.2 mg once a day

**Table 7:** Recommendations for peripheral μ-opioid receptor antagonists to treat opioid-induced constipation by various academic societies

Name of academic society (year)	Recommendation
American Gastroenterological Association (2019)	Naldemedine is recommended for opioid-induced constipation that is resistant to laxatives, rather than no treatment. (Level of evidence: high, strength of recommendation: strong) Naloxegol is recommended for opioid-induced constipation that is resistant to laxatives, rather than no treatment. (Level of evidence: moderate, strength of recommendation: strong) Methyl-naltrexone is recommended for opioid-induced constipation that is resistant to laxatives, rather than no treatment. (Level of evidence: low, strength of recommendation: conditional)
European Society of Neurogastroenterology and Motility (2020)	Peripheral μ-opioid receptor antagonists have a motor-promoting effect via antagonism of the inhibitory effect of μ-opioid analgesics on gastrointestinal motility. They are effective for the management of opioid-induced constipation. (Level of evidence: high, strength of recommendation: strong)
Japanese Society for Palliative Medicine (2020)	Conditional use of peripheral μ-opioid receptor antagonists is recommended in cancer patients with opioid-induced constipation. (Level of evidence: moderate, strength of recommendation: weak)
American Society of Supportive Care for Cancer (2020)	In patients with opioid-induced constipation, peripheral μ-opioid receptor antagonists should always be considered. (Level of evidence: strong, recommendation: recommended for cancer patients) ・In patients with opioid-induced constipation that is not responsive to peripheral μ-opioid receptor antagonists, consider adding another laxative. (Level of evidence: low, recommendation: suggestion) Laxatives (or peripheral μ-opioid receptor antagonists) should always be prescribed for patients receiving opioid analgesics. (Level of evidence: low, recommendation: suggested)
Japanese Society of Gastroenterology (2023)	For patients with suspected opioid-induced constipation, osmotic laxatives, stimulant laxatives, naldemedine, or lubiprostone is effective. However, the choice of laxative should be considered in light of the individual patient's condition, taking into account factors such as safety, cost, and type of opioid being administered. (Level of evidence: low; recommendation: none)
Japan Society of Pain Clinicians (2024)	The use of peripheral μ-opioid receptor antagonists is recommended for opioid-induced constipation that does not improve with general laxatives. (Level of evidence: B, recommendation level: 1)

#### Clinical question 5-6: How is drowsiness caused by opioid analgesics managed [134]?

##### Summary statement

Drowsiness is more likely to occur at treatment initiation or dose increase, but it usually lessens within a few days as tolerance develops. Unlike for cancer pain, it is not desirable to increase the dose of opioid analgesics until drowsiness occurs. If a drug with a central inhibitory effect is administered, more drowsiness might occur; caution is required with any concomitant medications.

#### Clinical question 5-7: What adverse events are associated with long-term use of opioid analgesics [135–137]?

##### Summary statement

When high-dose opioid analgesics are used for a long period of time, opioid analgesic-induced hyperalgesia and gonadal dysfunction can occur. It is advisable to limit the dose and avoid long-term use.

### VI Discontinuation of treatment with opioid analgesics

#### Clinical question 6–1: When should treatment with opioid analgesics be discontinued [92, 106, 138, 139]?

##### Summary statement

During treatment with opioid analgesics, discontinuation should be considered if the pain does not lessen or QOL does not improve, the risks of treatment outweigh the benefits, or dependence or abuse is suspected.

#### Clinical question 6-2: How should opioid analgesics be reduced or discontinued? [108, 140, 141]]

##### Summary statement

When considering reducing or discontinuing treatment with opioid analgesics, reduce the dose gradually (every 2–4 weeks). When reducing the dose, shorten the interval between medical examinations to check for changes in lifestyle and mental state due to worsening pain and the appearance of withdrawal symptoms. Do not reduce the dose or discontinue treatment too quickly.

#### Clinical question 6-3: What are the characteristics of patients who are likely to be treated with opioid analgesics for a long time or at high doses [142–145]?

##### Summary statement

Patients who are likely to require prolonged treatment with opioid analgesics or at high doses are characterized by pain of unclear cause, obsession with pain, excessive expectations of treatment, diffuse (generalized) pain, and comorbid mental symptoms, such as depression and anxiety.

#### Clinical question 6-4: How can patients who have been treated with opioid analgesics for a long time or on a higher dose be managed [99, 137, 146–148]?

##### Summary statement

During the administration of opioid analgesics, it is necessary to regularly assess for risk factors of opioid analgesic-related complications.

If the benefits do not outweigh the risks, the drug should be tapered and discontinued.

### VII. Inappropriate use of opioid analgesics

#### Clinical question 7-1: What is abuse of opioid analgesics [149]?

##### Summary statement

Abuse of opioid analgesics is a behavior or condition in which opioid analgesics are used for purposes other than treatment, such as to obtain a psychological effect such as euphoria.

#### Clinical question 7-2: What is physical dependence on opioid analgesics [149]?

##### Summary statement

Physical dependence on opioid analgesics is revealed by the development of drug-specific withdrawal symptoms due to the abrupt discontinuation of opioid analgesics, rapid dose reduction, or administration of an antagonist. Physical dependence is a physiological state of the body's adaptation to opioid analgesics.

#### Clinical question 7-3: What is the nature of psychological dependence on opioid analgesics [149, 150]?

##### Summary statement

Psychological dependence on opioid analgesics is a disorder of control over the use of opioid analgesics that is accompanied by a very strong desire (craving) for opioid analgesics or their psychological effects.

#### Clinical question 7-4: What are the symptoms of opioid withdrawal?

##### Summary statement

Opioid analgesic withdrawal syndrome occurs with abrupt reduction or discontinuation of opioid analgesics. Symptoms include unpleasant feelings, cravings for opioid analgesics, anxiety, nausea and vomiting, abdominal cramps, muscle pain, yawning, sweating, hot flashes, chills, lacrimation, nasal discharge, hypersomnia or insomnia, diarrhea, hair raising, and pupil dilation. These symptoms can also occur with therapeutic doses or the administration of opioid antagonists.

#### Clinical question 7-5: What are the characteristics of patients who are prone to inappropriate use of opioid analgesics [151]?

##### Summary statement

Characteristics of patients who are prone to inappropriate use of opioid analgesics during treatment include a history of substance use disorder, comorbid psychiatric disorders, and physical dysfunction due to pain. It is important to determine the indication for opioid analgesics after assessing a patient’s background and a thorough evaluation of risk factors.

#### Clinical question 7-6: How should inappropriate use of opioid analgesics be assessed [152, 153]?

##### Summary statement

For patients undergoing treatment with opioid analgesics, it is important to regularly evaluate the efficacy of the treatment and appropriate use, and to carefully and continuously monitor for any characteristic signs that suggest inappropriate use of opioid analgesics, such as dependence or abuse.

#### Clinical question 7-7: How should patients who have been using opioid analgesics inappropriately be managed [154]?

##### Summary statement

If inappropriate use of opioid analgesics is suspected, determine the severity of the inappropriate use and initiate appropriate actions. In Japan, there are no specialized treatment systems for psychological dependence on opioids, so it is important to prevent inappropriate use and address the situation as early as possible to prevent it from worsening into a serious condition.

#### Clinical question 7-8: How is opioid analgesic overdose (respiratory depression) treated [155]?

##### Summary statement

Overdose of opioid analgesics involves a rapid increase in blood concentration due to the ingestion of an intoxicating amount of opioid analgesics over a short period of time. The three major symptoms are miosis (pinpoint pupils), altered level of consciousness, and respiratory depression. Respiratory arrest from respiratory depression leads to death. When opioid analgesic overdose is suspected, the patient should first be fitted with electrocardiogram (ECG) and oxygen saturation (SpO_2_) monitoring, given oxygen, and have an intravenous line secured. Next, the μ-opioid receptor antagonist naloxone hydrochloride should be administered. The typical intravenous dose is 0.2 mg. If the effect is insufficient, additional 0.2 mg should be administered every few minutes. In case of a serious condition, advanced cardiac life support should be given first.

### VIII. Indications for treatment with opioid analgesics

#### Clinical question 8-1: Is treatment with opioid analgesics effective for chronic low back pain [156]?

##### Recommendation

Although non-invasive treatments are recommended as the first-line approach for chronic low back pain, we weakly recommend the use of opioid analgesics in patients with chronic low back pain who report severe pain when non-opioid medications are ineffective or contraindicated.**【2C】**

##### Summary statement

Although non-invasive treatments are recommended as the first-line approach for chronic low back pain, opioid analgesics may be considered for patients with severe chronic pain when other treatments prove ineffective or are contraindicated. While opioid analgesics can provide short-term pain relief and improve physical function, there is no evidence supporting their long-term use.

#### Clinical question 8-2: Is treatment with opioid analgesics effective for osteoarthritis (OA) [157–159]?

##### Recommendation

We weakly recommend the use of weak opioid analgesics for patients with OA with severe pain when non-opioid analgesics are ineffective or contraindicated. **[2B].**

##### Summary statement

Weak opioid analgesics may be considered for patients with OA who complain of severe pain and for whom non-opioid analgesics are ineffective or contraindicated. Although the use of weak opioid analgesics might improve analgesic efficacy and quality of life, adverse events are frequent, and extreme caution is required. On the other hand, strong opioid analgesics should not be readily used. Other treatment methods, including surgical therapy, should be thoroughly considered and the use of strong opioid analgesics should be considered only when the benefits are expected to outweigh the side effects. The treatment goals and risk of side effects should be fully explained to the patient before use.

#### Clinical question 8-3: Is treatment with opioid analgesics effective for compression fracture pain [160]?

##### Summary statement

Evidence for the use of opioid analgesics for compression fractures is lacking.

Short-term prescriptions for acute pain have been used, but the evidence for this is lacking.

#### Clinical question 8-4: Is treatment with opioid analgesics effective for herpes zoster-related pain [161, 162]?

##### Recommendation

We weakly recommend the use of opioid analgesics for herpes zoster-related pain. **[2D]**

##### Summary statement

Herpes zoster-related pain is typically due to acute inflammatory pain. In the chronic phase, neuropathic pain (i.e., post-herpetic neuralgia, PHN) can occur. Although opioid analgesics have a certain degree of efficacy against herpes zoster-related pain, there are many aspects of their long-term efficacy and adverse effects that have not been proven. High-quality randomized-controlled trials (RCTs) are desired in the future.

#### Clinical question 8-4-1: Is tramadol effective for post-herpetic neuralgia (PHN) [25, 163, 164]?

##### Recommendation

We weakly recommend the use of tramadol for PHN.** [2C].**

##### Summary statement

Tramadol may be useful for PHN. The evidence is weak, but its use is not ruled out.

#### Clinical question 8-4-2: Are strong opioid analgesics effective for post-herpetic neuralgia (PHN) [165, 166]?

##### Recommendation

We weakly recommend the use of strong opioid analgesics for PHN.** [2D]**

##### Summary statement

Although strong opioid analgesics might be useful for PHN in the short term, there are only small-scale, short-term RCTs. Their long-term usefulness and adverse effects are not clear.

#### Clinical question 8-5: Is treatment with opioid analgesics effective for painful diabetic neuropathy (PDN) [167–179]?

##### Recommendation

We weakly recommend the use of tramadol for PDN.** [2D]**

##### Summary statement

Pregabalin, mirogabalin, tricyclic antidepressants, duloxetine, aldose reductase inhibitors, mexiletine, and tramadol are recommended for the treatment of PDN. Concurrent treatment for diabetes is required. Regarding opioid analgesics other than tramadol, there have been RCTs of oxycodone extended-release formulations in recent years, but there is insufficient evidence regarding the benefits and harms associated with long-term use. The use of opioid analgesics other than tramadol requires careful consideration by pain specialists.

#### Clinical question 8-6: Is treatment with opioid analgesics effective for chronic post-surgical pain (CPSP) [180–184]?

##### Summary statement

There are reports on the use of pregabalin, gabapentin, and ketamine in the pharmacological treatment of CPSP. Although there are only a limited number of RCTs, pregabalin has been shown to be effective. Prolonged use of inappropriate opioid analgesics after surgery carries the risk of transitioning to chronic use, so the indiscriminate use of opioid analgesics for CPSP is not recommended. Although appropriate individualized measures are necessary, the need for opioid analgesics should be assessed. The dose should be reduced or the program should be terminated promptly.

#### Clinical question 8-7: Is treatment with opioid analgesics effective for post-spinal cord injury pain? [185, 186]

##### Summary statement

Gabapentinoids and antidepressants are recommended for neuropathic pain in the chronic phase after spinal cord injury. There is no evidence regarding the efficacy and toxicity of opioid analgesics for this condition. On the other hand, there are reports of long-term use of opioid analgesics at high doses, so care is needed regarding the dose and duration.

#### Clinical question 8-8: Is treatment with opioid analgesics effective for phantom limb pain (PLP) [187]?

##### Summary statement

Gabapentin and pregabalin are expected to be effective in the treatment of neuropathic pain associated with PLP in the chronic phase. Although there are reports that short-term opioid analgesics such as morphine have been effective in reducing the intensity of PLP, there is not enough evidence for a long-term effect. Caution should be exercised with regards to adverse effects.

#### Clinical question 8-9: Is treatment with opioid analgesics effective for complex regional pain syndrome (CRPS) [188]?

##### Summary statement

There is no evidence for the long-term efficacy of opioid analgesics in CRPS. Instead, caution should be exercised with regard to adverse effects.

#### Clinical question 8-10: Is treatment with opioid analgesics effective for thalamic pain?

##### Summary statement

Thalamic pain mainly occurs when the somatosensory cortex centered on the thalamus is damaged due to a stroke; it often appears during the recovery period for a stroke. Since thalamic pain is a symptom from which it is difficult to recover, long-term use of opioid analgesics is considered to be associated with many problems and is not recommended.

#### Clinical question 8-11: Is treatment with opioid analgesics effective for chemotherapy-induced peripheral neuropathy (CIPN) pain [189–195]?

##### Summary statement

CIPN is a major clinical problem in patients with cancer. It affects their QOL long after chemotherapy has been completed. The European Society of Clinical Oncology, American Society of Clinical Oncology, Oncology Nursing Society, National Cancer Institute, and National Comprehensive Cancer Network, and many other societies have developed treatment guidelines for CIPN.

In Japan, the Japanese Association of Supportive Care in Cancer has developed treatment guidelines. There is no evidence that opioid analgesics prevent or treat CIPN.

#### Clinical question 8-12: Are there conditions for which opioid analgesics are not recommended in principle [88, 196, 197]?

##### Summary statement

The prescription of opioid analgesics is considered to be extremely risky. Thus, it is not recommended for patients with a history of mental illness, substance use disorders such as alcohol abuse, sleep apnea syndrome, and serious internal organ diseases. They are also not recommended for pregnant women or young people.

### IX. Prescription of opioid analgesics in special circumstances

#### Clinical question 9–1: What are the considerations for prescribing opioid analgesics to pregnant patients [198]?

##### Summary statement

Although opioid analgesics do not affect fetal development, caution is required, because there is a risk of respiratory depression and withdrawal symptoms after birth.

#### Clinical question 9–2: What are the considerations for prescribing opioid analgesics to elderly patients [199, 200]?

##### Summary statement

The use of opioid analgesics in elderly patients should be approached with caution, because the risk of various adverse events is increased, which can lead to injury or death. Even when it is determined that opioid analgesics are necessary, both the initial and maintenance doses should be lower than those for younger patients.

#### Clinical question 9-3: What are the considerations for prescribing opioid analgesics to patients with impaired renal function [201]?

##### Summary statement

In patients with impaired renal function, it is preferable not to use morphine and codeine, because the accumulation of their metabolites might cause neurotoxicity.

Caution is required with tramadol and oxycodone because of the accumulation of the unchanged drug or its metabolites.

#### Clinical question 9-4: What are the considerations for prescribing opioid analgesics to patients with impaired liver function [202]?

##### Summary statement

Since many opioid analgesics are metabolized in the liver, patients with liver dysfunction require a reduction in dose or frequency of administration.

#### Clinical question 9-5: What are the considerations for prescribing opioid analgesics to patients with sleep apnea syndrome (SAS) [203, 204]?

##### Summary statement

Opioid analgesics can cause or worsen SAS by suppressing the respiratory center in the medulla oblongata. In addition, respiratory depression induced by opioid analgesics may be exacerbated by SAS.

#### Clinical question 9-6: What are the considerations for prescribing opioid analgesics to patients with work-related injuries and traffic accidents [205]?

##### Summary statement

When prescribing opioid analgesics, it is important to understand the psychosocial factors involved, because issues such as compensation and litigation (e.g., traffic accidents and work-related accidents) can affect pain.

#### Clinical question 9-7: What are the considerations for prescribing opioid analgesics to adolescent and young adults (AYAs) [206]?

##### Summary statement

Because AYAs are at high risk of developing dependence and abuse of opioid analgesics, opioid analgesics should be avoided for the treatment of chronic non-cancer pain in this patient population.

### X. Other

#### Clinical question 10-1: Is the combination of opioid analgesics and adjuvant analgesics effective [207–210]?

##### Summary statement

The combination of opioid analgesics and adjuvant analgesics, such as antidepressants and antiepileptic drugs, might enhance the analgesic effect. However, depending on the combination, there is also a possibility of more side effects. Therefore, these drugs should be administered with attention to the clinical symptoms.

#### Clinical question 10-2: What are the considerations for patients being treated with opioid analgesics who want to drive [211]?

##### Summary statement

The package insert for opioid analgesics states that “drowsiness, dizziness, and loss of consciousness may occur, so be careful not to allow patients taking this medicine to operate machinery that involves risks such as driving a car.” In addition, the Ministry of Health, Labour and Welfare has issued a notice to doctors and pharmacists to ensure that patients are fully informed of any precautions when prescribing or dispensing medicines that contain warnings such as “Do not drive” in the instructions for use.

We conclude that prescribers have a duty to explain to patients that they should avoid driving while taking opioid analgesics.

#### Clinical question 10-3: What are the considerations when traveling abroad during treatment with opioid analgesics [212]?

##### Summary statement

If a patient who is using a medical narcotic for therapeutic purposes is traveling abroad, they can carry the relevant medical narcotic with them when entering and leaving the country by obtaining permission from the director of the local welfare bureau in advance.

#### Clinical question 10-4: What are the limitations of the guidelines for prescribing opioid analgesics for chronic non-cancer pain [88, 94, 213, 214]?

##### Summary statement

In North America, where the rate of opioid analgesic prescriptions is extremely high, guidelines have been established for opioid analgesic prescriptions for chronic non-cancer pain, and a certain level of usefulness has been reported. In recent years, due to advances in cancer treatment, there have been reports of an increase in the use of opioid analgesics for chronic non-cancer pain as well as pain in cancer survivors.

However, opinions differ on the usefulness of both guidelines. Further observation and evaluation are needed.

## Summary of the guidelines

1. These guidelines are intended to promote the understanding and dissemination of appropriate treatment methods involving opioid analgesics for chronic non-cancer pain, including in cancer survivors.

2. The aim of treatment with opioid analgesics in chronic non-cancer pain is to alleviate the patient's pain and improve their QOL that has been reduced due to pain without worsening their QOL due to adverse events.

3. The treatment of chronic non-cancer pain with opioid analgesics in Japan is based on a completely different philosophy than the philosophy for treating direct cancer pain.

4. Adhere to the “Indications” and “Dosage and Administration” of each opioid analgesic and its generic equivalents approved in Japan.

5. Comply with the contents of the package inserts for opioid analgesics.

6. Treatment with opioid analgesics for chronic non-cancer pain is not indicated for all patients; it should be limited to situations that meet the following criteria.

(1) Clear organic cause of persistent pain.

(2) No effective pain relief measures other than treatment with opioid analgesics

(3) Understanding of the purpose of treatment with opioid analgesics.

(4) Good adherence to medication (able to comply with medication instructions).

(5) No history of substance or alcohol dependence.

(6) The organic factors of pain outweigh the psychosocial factors.

(7) Obtain informed consent after full explanation of the purpose of treatment, efficacy, and problems of treatment, and treatment duration with a view to reducing the dose or stopping the drug to the patient and family members prior to starting treatment with opioid analgesics for chronic non-cancer pain, with confirmation and consent forms necessary for prescribing medical narcotics.

7. When prescribing opioid analgesics, consider taking some kind of action against side effects.

8. It is recommended that the dose be ≤ 60 morphine milligram equivalents/day. It is strongly recommended that the upper limit be 90 morphine milligram equivalents/day (Refer to Table 3).

9. The standard duration of treatment is considered to be 3 months. The dosage should be reduced with consideration of discontinuation after a maximum of 6 months. This does not apply to tramadol, but always consider its necessity and avoid unnecessary long-term continuation.

10. For pain in cancer survivors that is not directly caused by cancer, such as pain after treatment or pain associated with complications or pre-existing conditions, these guidelines should be followed.
